# This moral coil: a cross-sectional survey of Canadian medical student attitudes toward medical assistance in dying

**DOI:** 10.1186/s12910-017-0218-5

**Published:** 2017-10-27

**Authors:** Eli Xavier Bator, Bethany Philpott, Andrew Paul Costa

**Affiliations:** Michael G. DeGroote School of Medicine, Waterloo Regional Campus, McMaster University, 10B Victoria St S, Kitchener, ON N2G 1C5 Canada

**Keywords:** Medical students, Medical education, Medical assistance in dying, Assisted suicide, Euthanasia, End-of-life, Attitudes and opinions

## Abstract

**Background:**

In February, 2015, the Supreme Court of Canada struck down the ban on medical assistance in dying (MAiD). In June, 2016, the federal government passed Bill C-14, permitting MAiD. Current medical students will be the first physician cohort to enter a system permissive of MAiD, and may help to ensure equitable access to care. This study assessed medical student views on MAiD, factors influencing these views, and opportunities for medical education.

**Methods:**

An exploratory cross-sectional survey was developed and distributed to medical students across all years of a three-year Canadian undergraduate medical program. The investigators administered the survey to participants during academic sessions from November to December, 2015. Analysis of the results included summary descriptive statistics, Pearson’s chi-square test of independence to identify differences between participants by year of study, logistic regression to identify factors that influence students’ stances on MAiD, and Wilcoxon signed rank test to measure changes in student support for MAiD and comfort discussing MAiD.

**Results:**

There were 405 participants for a response rate of 87%. The majority of students (88%) supported the Supreme Court’s decision, 61% would provide the means for a patient to end their life, and 38% would personally administer a lethal medication. Students who were more willing to provide the means for MAiD found medical education/clinical experience and patient autonomy to be important contributors to their stances on MAiD. Those students who were less willing to provide the means for MAiD found religious/spiritual beliefs and teachings, as well as concern about potential negative consequences, to be important contributors to their stances on MAiD. Educational training desired by participants included medicolegal (91%), communication skills (80%), technical skills (75%), and religious (49%).

**Conclusions:**

Medical students generally supported and would provide the means for MAiD to patients. They also indicated a desire for directed medical education on MAiD.

**Electronic supplementary material:**

The online version of this article (10.1186/s12910-017-0218-5) contains supplementary material, which is available to authorized users.

## Background

On February 6, 2015, the Supreme Court of Canada unanimously struck down the ban on medical assistance in dying (MAiD). In the legislation and throughout this manuscript, MAiD refers to the provision by a medical professional of the means for a patient to end their life, as opposed to the withdrawal of care. On June 17, 2016, Bill C-14 was passed and became the nationwide law permitting MAiD. This legislation applies to competent adults with grievous and irremediable medical conditions who make a voluntary request, and whose deaths are reasonably foreseeable. These criteria are open to interpretation as to who can receive medically-assisted death. The bill also places the responsibility for administering MAiD on physicians and nurse practitioners. While Bill C-14 establishes a theoretical framework for access to care, it is possible that disparities in access will exist based on practitioners’ willingness to provide MAiD.

Given the nationwide legislation legalizing MAiD, the authors of this manuscript believe that Canadians have a right to this service within the existing legal framework. One of the major factors in striving toward equitable access to care is to examine physicians’ willingness to provide assistance in dying. The majority of the Canadian public has been in favour of MAiD for decades [1]. Additionally, 16 % of all physicians (and 32% of family physicians) surveyed by the Canadian Medical Association in 2011 had received a request for MAiD in the previous five years [2]. This indicates a need for physicians who are knowledgeable about MAiD and end-of-life care. Upon graduation, current medical students will become the first cohort to enter a social, legislative, and medical climate permissive of assisted dying. It is therefore imperative to assess their views on the issue and their educational needs, as they may be influential in shaping access to assisted dying.

Our objective was to assess the views of medical students at a Canadian medical school toward assisted dying, the factors which influence their views, and opportunities for medical education.

## Methods

### Survey design

This was an exploratory cross-sectional study of the medical students of a Canadian undergraduate medical program. There were no previous Canadian studies on which to base a data collection instrument. Using the findings from a literature search, and following the principles of the tailored design method, we developed a sixteen-question survey instrument to explore student views around MAiD (Additional file 1) [3]. This instrument was pilot tested by a faculty member (palliative care physician) and seven other medical school staff to assure content and face validity, clarity, and ease of use. The final survey was approved for distribution by this institution’s Research Ethics Board.

### Setting and participants

Surveys were administered to medical students in all years of study in a single undergraduate medical program. This program has a three-year curriculum in which students train year-round and graduate with a medical degree (M.D.). There were no exclusion criteria for participation in this study. As an incentive, participants were entered into a draw for three FITBIT® activity trackers, one per year of study. Surveying was completed in November and December of 2015. This was after the Supreme Court of Canada struck down the ban on MAiD, but before the legislation permitting MAiD was passed.

First- and second-year students were in the pre-clerkship or non-clinical phase of their training. These students completed the survey before mandatory small group educational sessions. Third-year students were in the clerkship or clinical phase of their training. This group was surveyed before optional educational sessions, so fewer students were exposed to the survey compared to the first- and second-year students. In all years of study, at least one mandatory session on MAiD through the regular medical curriculum had been delivered prior to survey administration. The potential size of this sample was approximately 617 students.

### Variables

Demographic data included year of study, gender, spirituality, and anticipated medical specialty (this specific data point is not displayed here). These questions were presented in a multiple choice format.

Students were asked to identify whether they supported the Supreme Court of Canada’s decision to strike down the ban on MAiD. In follow-up, they were asked if they would participate in MAiD by providing the means for a patient to end their own life (e.g., via prescription for lethal medication), or by administering a lethal drug themselves. These questions were prefaced with the condition that the student would be deciding from the position of a practicing physician, and the patient would meet the legal criteria for assisted death and other palliative options would have been exhausted. Additional questions asked students to decide whether they supported MAiD for patients with non-terminal grievous illness or terminal illness complicated by mental health illness, whether patient preferences for MAiD should be included in advance care directives, and whether palliative care in Ontario is adequate. All of these questions were presented in a ‘yes,’ ‘no,’ ‘unsure’ format.

Students completed a free-form question asking which medical specialty should be responsible for administering MAiD.

A Likert-type scale allowed students to rank the importance of various factors to their stance on MAiD. The scale rankings were ‘not important,’ ‘slightly important,’ ‘fairly important,’ ‘important,’ and ‘very important.’ A second Likert-type scale assessed changes in students’ stances on MAiD from before their medical school training to the time of the survey administration. The scale rankings were ‘strongly oppose,’ ‘somewhat oppose,’ ‘neutral,’ ‘somewhat support,’ and ‘strongly support.’ A third Likert-type scale assessed changes in student comfort with discussing issues around MAiD from before their medical school training to the time of survey administration. The scale rankings were, ‘very uncomfortable,’ ‘somewhat uncomfortable,’ ‘neutral,’ ‘somewhat comfortable,’ and ‘very comfortable.’

Finally, students were asked which educational content they would find helpful in preparing for end-of-life and MAiD decision-making. Options included technical training, education about the doctrines of different religions, communication skills training, and medical-legal information.

### Data analysis

The data were transcribed into Microsoft Excel using read-aloud and visual checking methods to ensure accuracy. Analysis was conducted using GNU PSPP (version 0.10.2), an open-source statistical package, and statistical significance was set at *p* < 0.05 a priori. Missing data was handled via listwise deletion. Descriptive statistics were used to summarize the data. Differences between participants by year of study were assessed using Pearson’s chi-square test of independence. Logistic regression was used to compare an independent variable, factors influencing students’ stance on MAiD (ordinal data), with a dependent variable, students’ willingness to provide MAiD. The ‘yes’ and ‘no’ responses to the question ‘would you provide the means for MAiD’ were used while ‘unsure’ responses were omitted, making this a binary variable. The Wilcoxon signed rank test was used to measure changes in students’ support for MAiD, and comfort discussing MAiD, before medical school and at the time of survey administration.

## Results

Demographic data is presented in Table 1. There were 405 respondents out of 465 medical students approached, yielding a total response rate of 87.1%. This sample represents two-thirds of the entire medical student population at this institution. The three years of study had a comparable distribution of students by gender and spirituality. Pearson’s chi-square test of independence (not shown here) showed no significant differences across year of training in the willingness to administer or provide the means for MAiD, or in the importance of different factors, described later in this paper, that could influence one’s stance on MAiD.

**Table 1:** Medical student demographic data

Year of study		Year 1	Year 2	Year 3
Response rate % (Participants/Approached)		80% (156/195)	95% (190/200)	84% (59/70)
Gender % (N)	Female	55% (85)	61% (114)	52% (30)
	Male	45% (69)	39% (74)	48% (28)
Religion % (N)	Atheist	28% (42)	24% (44)	26% (15)
	Agnostic	22% (34)	26% (48)	26% (15)
	Spiritual but not religious	21% (32)	21% (39)	12% (7)
	Religious non-practicing	15% (22)	10% (18)	15% (9)
	Religious practicing	14%(21)	19% (36)	21% (12)
Stance on MAiD				
1 – strongly oppose 5 – strongly support	Mean (SD)	4.12 (1.02)	4.27 (0.99)	4.19 (0.85)

Table 2 summarizes student opinions surrounding MAiD and end-of-life issues. The vast majority of students (88%) supported the Supreme Court’s decision to allow MAiD, and many (61%) would provide the means for a patient to end their own life. Furthermore, 38% would personally administer a lethal medication. From a qualitative perspective, students tended to choose ‘unsure’ or ‘yes’ more than ‘no’ when asked whether they would personally administer MAiD, and whether MAiD should be an option for patients with grievous non-terminal illness or terminal illness complicated by mental illness. However, students were clearly in agreement that patients’ MAiD preferences should be included in advance care directives (82%), and that access to palliative care in Ontario is inadequate (73%).

**Table 2:** Medical student opinions on MAiD and end-of-life issues

Question	% (N)		
	No	Unsure	Yes
Is current access to palliative care in Ontario adequate?	73% (295)	23% (93)	4% (15)
Do you support the Supreme Court of Canada decision to allow MAiD?	5% (20)	7% (29)	88% (354)
Should MAiD be including in advance care directives (living wills) to guide treatment if the patient becomes unable to give consent?	8% (33)	10% (41)	82% (329)
If you were a practicing physician and a patient met the legal criteria for MAiD, after other palliative options were considered…			
Would you provide the means for a patient to end their own life if they requested it?	11% (45)	28% (112)	61% (246)
Would you personally administer a lethal dose of medication to assist a patient in ending their life if they requested it?	23% (93)	39% (158)	38% (153)
Do you support the provision of MAiD to someone suffering from a terminal illness that also has a mental health illness?	18% (75)	52% (209)	30% (121)
Do you support the provision of MAiD to someone suffering from an illness that is not terminal but has a severe negative impact on their quality of life?	16% (64)	42% (171)	42% (169)

Participants were asked to list the specialties they thought should be responsible for administering MAiD. Palliative care was the most chosen specialty by a large margin (33% of all responses), followed by family medicine (16%) and internal medicine (11%). Some students suggested that any specialty (5%) or a new specialty (2%) should administer MAiD.

Table 3 displays factors which students ranked on a Likert-type scale in terms of importance to their stances on MAiD. According to the median responses, students placed the most importance on patient autonomy. The least important factors were religious and spiritual beliefs or teachings, and personal experiences with the death of family or friends. Those who found patient autonomy and medical education/clinical experience to have an important influence on their stance were 2.81 and 1.64 times more likely to be willing to provide the means for MAiD, respectively. Conversely, those who placed more importance on religious/spiritual beliefs and teachings, and the potential for negative consequences secondary to MAiD, were 2.61 and 1.67 times less likely to be willing to provide the means for MAiD, respectively.

**Table 3:** Factors influencing students’ stances on MAiD

Factors influencing stance 1 – not important 5 – very important	Mean Response (SD)	Would you provide the means for a patient to end their own life if they requested it? (*p*-value)			
		Odds Ratio	*p* Value	Lower CI	Upper CI
Patient autonomy	4.57 (0.71)	+2.81	0.004	1.38	5.72
Patient diagnosis	4.25 (0.88)	+1.04	0.890	0.58	1.88
Knowledge about palliative care	4.15 (0.95)	−1.60	0.161	0.83	3.06
Legality of MAiD	4.13 (1.10)	+1.29	0.286	0.81	2.04
Potential for negative consequences (abuse, slippery slope)	3.77 (1.19)	−1.67	0.035	1.04	2.68
Personal perception of physician’s role	3.70 (1.06)	−1.64	0.062	0.98	2.76
Medical education/clinical experience	3.52 (1.11)	+1.64	0.029	1.05	2.57
Personal morals	3.50 (1.24)	−1.17	0.532	0.71	1.94
Patient age (child, middle aged, elderly)	3.39 (1.10)	+1.02	0.932	0.64	1.62
Opinion of medical community	3.15 (1.09)	+1.29	0.319	0.78	2.13
Experience with death of family/friend	2.48 (1.27)	+1.30	0.219	0.85	1.98
Religious/spiritual beliefs/teachings	2.29 (1.39)	−2.61	<0.001	1.70	4.01

(+) indicates increased likelihood while (−) indicates decreased likelihood

Medical students’ support for MAiD, as measured on a 5-point Likert-type scale, increased from a mean of 3.90 (SD = 1.06) before medical school to 4.20 (SD = 0.98) at their current level of training. The Wilcoxon signed rank test shows this to be a significant change (Z = −7.35, *p* < 0.001) with a small effect size of 0.26. There were no significant differences across year of training in terms of initial stance on MAiD and stance at current level of medical education. Comfort discussing the issues around MAiD also significantly increased from a mean of 3.31 (SD = 1.08) before medical school to 3.82 (SD = 0.89) at current level of training (Z = −9.35, *p* < 0.001, moderate effect size of 0.33).

Finally, students were asked about the kinds of education they wanted to prepare for MAiD. In order of preference, 91% of students wanted medical-legal training, 80% wanted communication skills training, 75% wanted training on the technical aspects of administering MAiD, and 49% wanted education around the doctrines of different religions. Although freeform comments were not formally analyzed, some participants expressed a desire for more clinical exposure to end-of-life care and MAiD.

## Discussion

This, to our knowledge, is the first Canadian study to assess MAiD from a medical student perspective. The primary objective was to identify medical student opinions about medical assistance in dying, the factors influencing their views, and opportunities for medical education. Most medical students across all years of study were supportive of MAiD, and a majority would be willing to provide patients with the means for assisted dying in the appropriate circumstances. The factor most important to students’ stances on MAiD was the principle of patient autonomy. The least important factors were religious/spiritual beliefs and teachings, and personal experiences with death. The majority of students desired medical-legal, communication skills, and technical training specific to MAiD.

Medical students in this sample were generally supportive of MAiD. However, responses were mixed when students were faced with specific scenarios like the provision of MAiD in the context of mental health illness or non-terminal illness with poor quality-of-life. Specific patient factors may influence decision-making on a case-by-case basis, making broader questions harder to answer definitively. Comparing the results of this study with others is difficult considering the unique contexts within which each study was conducted [4–11]. However, medical students in our study sample were more supportive of MAiD than Canadian physicians have been in previous research, and this phenomenon has been noted in studies conducted outside of Canada [7, 9, 10]. The Canadian Medical Association conducted a poll of 2125 members in 2011, before the ban on MAiD was struck down, and found that 20% would participate in MAiD were it legalized [2]. A follow-up poll of 1407 members was conducted in mid-2015, months after the Supreme Court of Canada struck down the ban on MAiD [12]. This poll demonstrated only modest change; only 29% would consider providing MAiD, while 63% would refuse outright. The results collected by the Canadian Medical Association may be affected by response bias, but suggest that practicing physicians continue to be less supportive of MAiD despite the changing legal climate.

Students in this study acknowledged an inadequacy of access to palliative care. Approximately 95% of Canadians would benefit from palliative services at the end-of-life, but about 70% lack access secondary to uneven distribution of sparse services [1]. We also found that our sample most often selected palliative care physicians to be responsible for providing MAiD over other specialties. It has been argued that since palliative care specialists are trained to deliver comprehensive end-of-life care, they are the best positioned to deliver MAiD synergistically with other palliative services [13–15]. However, 56% of members of the Canadian Society of Palliative Care physicians have stated that MAiD does not fall within their role, and internationally, palliative physicians have generally shown opposition to MAiD [9, 16–19].

Factors that influence the stance of medical professionals in regards to MAiD have been extensively studied [7, 10, 16, 20–23]. Our study contributes to the existing literature that those who were willing to provide the means for MAiD to patients were more likely to place more importance on patient autonomy and medical education when choosing their position on MAiD. Conversely, those who were less willing to provide the means for MAiD were more likely to place importance on religion and spirituality, as well as the potential for negative consequences. Other factors identified in the literature as predictors of increased support for MAiD include wishes to provide death with dignity and the relief of suffering, as well as the avoidance of futile treatments [16, 23]. Factors identified in the literature as predictors of decreased support for MAiD aside from religiosity include involvement in the care of the dying; employment in rural towns; and the emphasis of palliative care as a substitute for MAiD, of difficulties in predicting life expectancy, of legal factors, and of the potential for abuse secondary to external pressures [16, 22, 23].

The majority of medical students in our study desired medical-legal, communication skills, and technical training to prepare them for MAiD. Historically, medical students have rated their exposure to end-of-life care and ethical decision-making as inadequate, although this may not represent current medical education [5, 21]. Factors shown to improve medical student preparedness for end-of-life discussions include educational exposures and positive role models [24]. The students in our study became more comfortable discussing the issues around MAiD as they progressed through their medical education. They also displayed a more positive self-rated stance toward MAiD with more training, similar to the attitude of the Canadian public and dissimilar to the attitude of practicing physicians. Students have previously displayed more comfort with complex ethical decision-making with directed training [5, 21]. However, the literature suggests that specific MAiD programming may not significantly impact students’ views on MAiD. Students’ pre-formed opinions on end-of-life issues like withdrawal of treatment and MAiD have been found to remain unchanged after directed educational programs [5, 6, 21]. It is possible that the ethical curriculum at the institution studied here may present MAiD through a more positive lens than other medical schools, but this cannot be determined without further research, especially when this study was conducted in the midst of changing legal circumstances.

### Limitations

Our study sample included students from a single medical school. Therefore, caution should be exercised when generalizing these results. Nevertheless, our response rate lends strength to our findings. Due to limited resources and access to the population, it was not possible to reassess medical student attitudes longitudinally throughout medical school or residency. The three cohorts at different stages in their training showed no significant differences in demographics or opinions, although it would have been very interesting to see if student opinions would change after MAiD became legal in 2016. In terms of the survey instrument, a ‘depends on the specific context’ response option may have improved the quality of the data for some questions. Furthermore, the survey question addressing MAiD in the context of mental illness could be improved by identifying specific medical conditions, and clarifying whether they were treated at the time where MAiD is being considered. Our list of factors influencing student views on MAiD was also not exhaustive, and we could have presented more nuanced distinctions between different ethical principles and more precise definitions.

## Conclusions

Canadian medical students in this study are supportive of the practice of MAiD and a majority would provide this service. Their stances are influenced by multiple factors, and their willingness to participate in MAiD is impacted by concerns about patient autonomy, medical education, religion and spirituality, and the potential for negative consequences. A majority of these students want medical-legal, communication skills, and technical training centred on MAiD. Areas for future research may include the assessment of theoretical and practical education programs that provide exposure to MAiD, the inclusion of MAiD in advance care directives (which was largely supported by our sample), the evaluation of patient access to MAiD, and the relationship between MAiD and palliative services in practice. As the number of currently practicing physicians willing to provide MAiD may limit access to the service, it is vital that medical education exposes trainees to ethical decision-making around, and compassionate delivery of, MAiD.

## Additional file

Additional file 1: Survey of medical student attitudes toward physician-assisted death. (PDF 136 kb)

## Abbreviation

MAiD: Medical assistance in dying
