# Supplementary material for: This moral coil: a cross-sectional survey of Canadian medical student attitudes toward medical assistance in dying
Source: BMC Med Ethics. 2017 Oct 27;18:58. doi: 10.1186/s12910-017-0218-5 (PMC5658957; doi:10.1186/s12910-017-0218-5)
Supplement: Additional file 1: — Survey of medical student attitudes toward physician-assisted death. (PDF 136 kb) [file 12910_2017_218_MOESM1_ESM.pdf]

**ADDITIONAL FILE 1**

## Survey of Medical Student Attitudes Toward Physician-Assisted Death

In February 2015, the Supreme Court of Canada unanimously struck down the ban on physician-assisted death. Within the next year, assisted dying in some form will become an option for competent, consenting adults who suffer from “a grievous and irremediable medical condition.” The goal of this survey is to assess the attitudes of medical students toward physician-assisted death and the factors which influence their decision making. Your input is very much appreciated!

---

**PLEASE CIRCLE GRADUATING YEAR: 2016 2017 2018**

---

1. Do you support the Supreme Court of Canada decision to allow physician-assisted death?
    a) Yes
    b) No
    c) Undecided

---

If you were a practicing physician, and if a patient met the legal criteria for physician assisted death after other palliative treatment options had been considered...

2. Would you provide the means for a patient to end their own life if they requested it (e.g., a prescription for a lethal medication)?
    a) Yes
    b) No
    c) Undecided

3. Would you personally administer a lethal dose of medication to assist a patient in ending their life if they requested it?
    a) Yes
    b) No
    c) Undecided

---

4. Do you support the provision of assisted death to someone suffering from a terminal illness who also has mental health illness (e.g. depression)?
    a) Yes
    b) No
    c) Undecided

---

5. Do you support the provision of assisted death to someone suffering from an illness that is not terminal but has a severe negative impact on the person’s quality of life?
    a) Yes
    b) No
    c) Undecided

---

-----------------------------------------------------------------------------------------------------------

6. Should physician-assisted death be included in advance care directives (living wills) to guide treatment if the patient becomes unable to give consent?
    a) Yes
    b) No
    c) Undecided

-----------------------------------------------------------------------------------------------------------

7. What medical specialty(ies) do you feel should be responsible for providing physician-assisted death?

Please list: ______________________________________________

-----------------------------------------------------------------------------------------------------------

8. How important are the following factors to **your** stance on physician-assisted death as an option for patients who request it?

| | Not Important | Slightly Important | Fairly Important | Important | Very Important |
|---|---|---|---|---|---|
| a) Legality of physician-assisted death | ☐ | ☐ | ☐ | ☐ | ☐ |
| b) Opinion of medical community | ☐ | ☐ | ☐ | ☐ | ☐ |
| c) Personal perception of physician's role | ☐ | ☐ | ☐ | ☐ | ☐ |
| d) Patient diagnosis | ☐ | ☐ | ☐ | ☐ | ☐ |
| e) Patient autonomy | ☐ | ☐ | ☐ | ☐ | ☐ |
| f) Patient age (child, middle-age, elderly) | ☐ | ☐ | ☐ | ☐ | ☐ |
| g) Personal morals | ☐ | ☐ | ☐ | ☐ | ☐ |
| h) Religious or spiritual beliefs/teachings | ☐ | ☐ | ☐ | ☐ | ☐ |
| i) Experience with death of family/friend | ☐ | ☐ | ☐ | ☐ | ☐ |
| j) Medical education/clinical experience | ☐ | ☐ | ☐ | ☐ | ☐ |
| k) Knowledge about palliative care | ☐ | ☐ | ☐ | ☐ | ☐ |
| l) Potential for negative consequences (e.g., abuse, slippery slope argument) | ☐ | ☐ | ☐ | ☐ | ☐ |

9. Are there any other factors that influence your stance on physician-assisted death?

Please list: ______________________________________________

-----------------------------------------------------------------------------------------------------------

10. Do you believe that current access to palliative care in Ontario is adequate?
    a) Yes
    b) No
    c) Undecided

-----------------------------------------------------------------------------------------------------------

---

11. Has your stance on the principle of physician-assisted death changed as a result of your medical school education and clinical experience?

| | Strongly Oppose | Somewhat Oppose | Neutral | Somewhat Support | Strongly Support |
|---|---|---|---|---|---|
| a) Before medical education | ☐ | ☐ | ☐ | ☐ | ☐ |
| b) At current level of training | ☐ | ☐ | ☐ | ☐ | ☐ |

---

12. Has your comfort level in considering the issues around physician-assisted death changed as a result of your medical school education and clinical experience?

| | Very Uncomfortable | Somewhat Uncomfortable | Neutral | Somewhat Comfortable | Very Comfortable |
|---|---|---|---|---|---|
| a) Before medical education | ☐ | ☐ | ☐ | ☐ | ☐ |
| b) At current level of training | ☐ | ☐ | ☐ | ☐ | ☐ |

---

13. What content would you find helpful in your formal medical education to prepare you for end-of-life and assisted death decisions? Select all that apply.
    a) Technical training
    b) Education about the doctrines of different religions
    c) Communication skills training
    d) Medical-legal information
    e) Other (please specify):________________________________________

---

14. How do you identify? You may select multiple options.
    a) Woman/female
    b) Man/male
    c) Neither male/female
    d) Intersex
    e) Transgender

---

15. What would best describe your beliefs system? You may select multiple options.
    a) Religious and practicing
    b) Religious and non-practicing
    c) Spiritual but not religious
    d) Agnostic
    e) Atheist

---

---

16. What is your anticipated specialty, if known? You may select multiple options.
    a) Anesthesiology
    b) Emergency medicine
    c) Family medicine
    d) Internal medicine
    e) Laboratory medicine
    f) Obstetrics and gynecology
    g) Oncology
    h) Palliative Care
    i) Pediatrics
    j) Psychiatry
    k) Radiology
    l) Surgery and subspecialties
    m) Undecided
    n) Other (please specify): ________________________________________

---

PLEASE SHARE ANY ADDITIONAL COMMENTS BELOW:

Do you have any answers you wish to clarify?
Is there any specific medical education/exposure you would like?
What would help you to change/clarify your stance of physician-assisted death?

____________________________________________________________

____________________________________________________________

____________________________________________________________

____________________________________________________________

____________________________________________________________

____________________________________________________________

____________________________________________________________

____________________________________________________________

____________________________________________________________

____________________________________________________________

____________________________________________________________
